# The research topic landscape in the literature of social class and inequality

**DOI:** 10.1371/journal.pone.0199510

**Published:** 2018-07-02

**Authors:** Liang Guo, Shikun Li, Ruodan Lu, Lei Yin, Ariane Gorson-Deruel, Lawrence King

**Affiliations:** 1 Institute of Computational Social Science, Shandong University, Weihai, China; 2 Engineering Department, Cambridge University, Cambridge, United Kingdom; 3 Institut Supérieur de Management et Communication, Paris, France; 4 Kantar TNS, Paris, France; 5 Department of Economics, University of Massachusetts, Amherst, MA, United States; CUNY, UNITED STATES

## Abstract

The literature of social class and inequality is not only diverse and rich in sight, but also complex and fragmented in structure. This article seeks to map the topic landscape of the field and identify salient development trajectories over time. We apply the Latent Dirichlet Allocation topic modeling technique to extract 25 distinct topics from 14,038 SSCI articles published between 1956 to 2017. We classified three topics as “hot”, eight as “stable” and 14 as “cold”, based on each topic’s idiosyncratic temporal trajectory. We also listed the three most cited references and the three most popular journal outlets per topic. Our research suggests that future effort may be devoted to Topics “urban inequalities, corporate social responsibility and public policy in connected capitalism”, “education and social inequality”, “community health intervention and social inequality in multicultural contexts” and “income inequality, labor market reform and industrial relations”.

## Introduction

Social stratification or social class refers to visible societal layers or classes of differing wealth, income, race, education or power [1]. Social stratification, social class and social inequality (hereafter social class and inequality) are often used interchangeably, all of which are the products of an unequally structured society in which identities are socially produced on a large scale [2]. As societies evolve, the number of layers can change, and the boundaries between them move. Mobility within and between classes and their persistence from one generation to another influences a society’s governance, customs, culture, identity, and social inequality perception [3]. Recent so-called “black swan events” (i.e. Donald Trump’ victory in the American election and the Brexit referendum) and the growth of populism in Europe are the vivid examples of how human society is transformed by the struggle between different social classes.

Social scientists have studied social class and inequality at length. In the 19th century, Marxian theories of stratification [4] considered social inequality as crucial to understand human society. The struggle between the exploited and exploiting classes would eventually lead to a political revolution, which would replace private monopolies by total equality (e.g. the Soviet Union and Communist China). In the early 20th century, Max Weber proposed the three-component theory of stratification, with class, status and power as distinct ideal types and social class manifests itself as unequal access to economic resources [5] In the late 20th century, Lenski [6] developed the theory of social stratification, further arguing that the accumulation of information, especially technological information, is the most basic and powerful factor in the evolution of human societies. Technological advances laid the foundations for social inequality in terms of power and wealth distribution.

Based on classic social theories, many studies have empirically examined the determinants and consequences of social class and inequality. Multidisciplinary knowledge in the field is not only diverse and insightful, but also fragmented and multifaceted. There is a pressing need for clear mapping of this ever more complex landscape to help researchers and students to conduct efficient, effective literature reviews. A comprehensive mapping of the field will help by providing an understanding of how it has evolved over time, shedding light on the points of consensus and divergences among scholars, while revealing research gaps in the intellectual structure of the field.

This study comprises a computer-based overview of the social class and inequality literature over the period of 1956–2017. First, we mapped out the topic landscape, and then attempted to anticipate hot topics that will generate seminal research in the future. As far as we know, this is the first systematic review of the field across many disciplines over seven decades and the first attempt to forecast topic prevalence in this literature. Our first contribution lies in uncovering a hidden structure of 25 distinct topics and development trajectories in a corpus comprising the abstracts of 14,038 scholarly articles. This study draws on an unprecedentedly large text corpus that includes a broad range of author backgrounds, disciplinary influences and research focuses. Our study will enable researchers to explore not only topic development paths within the overall literature, but also the most salient articles in each individual topic. Our second contribution lies in forecasting the popularities of these 25 topics, based on each topic’s temporal idiosyncrasies which will help both researchers and journal editors to select promising research topics. In the next section, we briefly introduce topic modeling techniques and applications in modeling scientific literature. Then we describe our analyses and results. And finally, we discuss the implications of our work for scholars, journal editors, and practitioners.

## Topic modeling methodology

A document can be represented as a vector of word term weights (i.e. features) from a set of terms (i.e. dictionary) and the topic of a document is made of a joint membership of terms which have a pattern of occurrence [7]. Early document clustering techniques employ the vector space modeling technique, which can calculate the similarity between two documents [8]. This technique fails to deal with the issues caused by synonymy (i.e. different words with similar or identical meanings) and polysemy (i.e. the words with different meanings in different contexts). Later, Latent Semantic Analysis (LSA) was developed in an effort to improve classification performance in document retrieval [9]. Like most topic modeling techniques, LSA starts from a pre-processing step, which cleans the corpus of a set of text documents and builds a document-term matrix for subsequent modeling. The cleaning procedures include tokenization (i.e. partitioning a text document into a list of tokens), stop-word removal (i.e. removing the words that are extremely common but are of little value in helping classifying documents, such as this, it, is), stemming and lemmatization (i.e. removing the ends of conjugated verbs or plural nouns while keeping the lemma, base or root form), and compound words (i.e. concatenating hyphenated words that describe one concept). The remaining words are used to construct a document-term-matrix (DTM). The DTM is a matrix where each row represents a document, each column represents a unique word, and each cell denotes the number of times a given word appears in a given document. Then, LSA reduces the DTM into a filtered DTM through singular value decomposition (SVD). Finally, LSA computes the similarity between text documents to pick the heist efficient related words. While computationally efficient, LSA fails to identify and distinguish between different contexts of word usage without recourse to a dictionary or thesaurus [10].

Backed by Bayesian statistics, Latent Dirichlet Allocation (LDA) is developed to apply a probabilistic model to analyze word distributions in text documents and uncover topics in an automated fashion [7,11]. This generative modeling technique does not require prior categorization, labelling and annotation of the texts but reveals the invisible, latent topic structure through statistical procedures [12]. Instead, it follows the “bag-of-words” assumption to treat a document as a vector containing the count of each word type, regardless the order in which they appear. In a nutshell, LDA assumes that each document can be modelled as a mixture of topics, and each topic is a discrete probability distribution that defines how likely each word is to appear in a given topic. A document is then represented by a distribution of topic probabilities. It estimates the parameters in the distributions of word and of topics with Markov chain Monte Carlo (MCMC) simulations [7]. LDA then assigns topics to each document through a Dirichlet distribution of topics. Given a specific number of topics in a collection of text documents, the extent to which each topic (and its associated words) is represented in a specific document can be modelled by a latent variable model, where latent variables represent the topics and how each document in the collection manifests them [7,13]. In short, LDA discovers patterns of word use and connect patterns of similar use to estimate the posterior distribution of hidden variables, which represents the topic structure of the collection [12,13].

Recently, some LDA-based techniques have been proposed. For example, Correlated-Topic-Model (CTM) uses a logistic normal distribution to create relations among topics [13]. Supervised LDA [14] can introduce known label information into the topic discovery process. Labeled LDA (LLDA) [15] allows for multiple labels of documents and for the relation of labels to topics represents one-to-one mapping. Partially labeled LDA (PLLDA) [16] further extends LLDA to have latent topics missing from the given document labels.

LDA has been widely used to process otherwise unmanageably large volumes of text, identify the most salient topic in a single document, investigate similarities between documents, and uncover topic prevalence over time [11,13,17]. We summarize some recent applications of LDA in scientific topic discovery in Table 1.

**Table 1:** A non-exhaustive list of LDA applications in scientific topic discovery.

Articles	Research Areas
Heo, Kang, Song, & Lee [40]	Biology
Karami, Gangopadhyay, Zhou, & Kharrazi [41]	Computer Science
Figuerola, Marco, & Pinto [42]	
Yau, Porter, Newman, & Suominen [43]	
Hu, Fang, & Liang [44]	
Das, Sun, & Dutta [45]	Civil Engineering
Westgate, Barton, Pierson & Lindenmayer [46]	Environmental Sciences
Tvinnereim & Flottum [47]	
Carnerud [48]	Management
Antons et al. [12]	
Farrell [49]	Political Science
Bittermann & Fischer [50]	Psychology
Oh, Stewart, & Phelps [51]	
Wang, Ding, Zhao, Huang, Perkins, Zou & Chen [52]	Public, Environmental & Occupational Health
Sun & Yin [53]	Transportation Science & Technology

### Description of the sample

We extracted article abstracts from the core collection of the Web of Science (WoS) database using the following criteria: articles published in English, whose topic terms (i.e. titles, abstracts and keywords) included “social stratification(s)”, “social class(es)” or “social inequality(ies)” in SSCI indexed journals over the period of 1956 to December 2017. The search found 15,057 articles. We deleted those without keywords and abstracts, leaving 14,038 articles in the collection. Among these articles, 67.11% belong to “social class(es)” alone, 23.60% to “social inequality(ies)” alone and 6.71% to “social stratification(s)” alone. There are 1.74% of articles that belong to both “social class(es)” and “social inequality(ies)”; 0.52% to “social class(es)” and “social stratification(s)”; and 0.26% to both “social inequality(ies)” and “social stratification(s)”. There are only 0.04% of articles that belong to three topic terms.

In addition, we built three time series in terms of annual article counts for these three terms respectively. The correlation coefficients between “social class(es)” and “social inequality(ies)” series is 0.87, between “social class(es)” and “social stratification(s)” series is 0.86, and between “social inequality(ies)” and “social stratification(s)” series is 0.97. These statistics confirm that the three topic themes are highly similar. They all reflect the types of social divisions envisaged by Marx and refer to groups defined by their relationship to ownership and control over the means of production, of labor and of distribution [18]. We did not include the term “social status” because it emphasizes the social distinctions caused not only by economic factors but also by cultural ones, which include denotative (what is), normative (what should be), and stylistic (how done) beliefs, shared by a group of individuals who have undergone a common historical experience and participate in an interrelated set of social structures [19].

## Analyses and results

### Descriptive statistics

**Fig 1:**
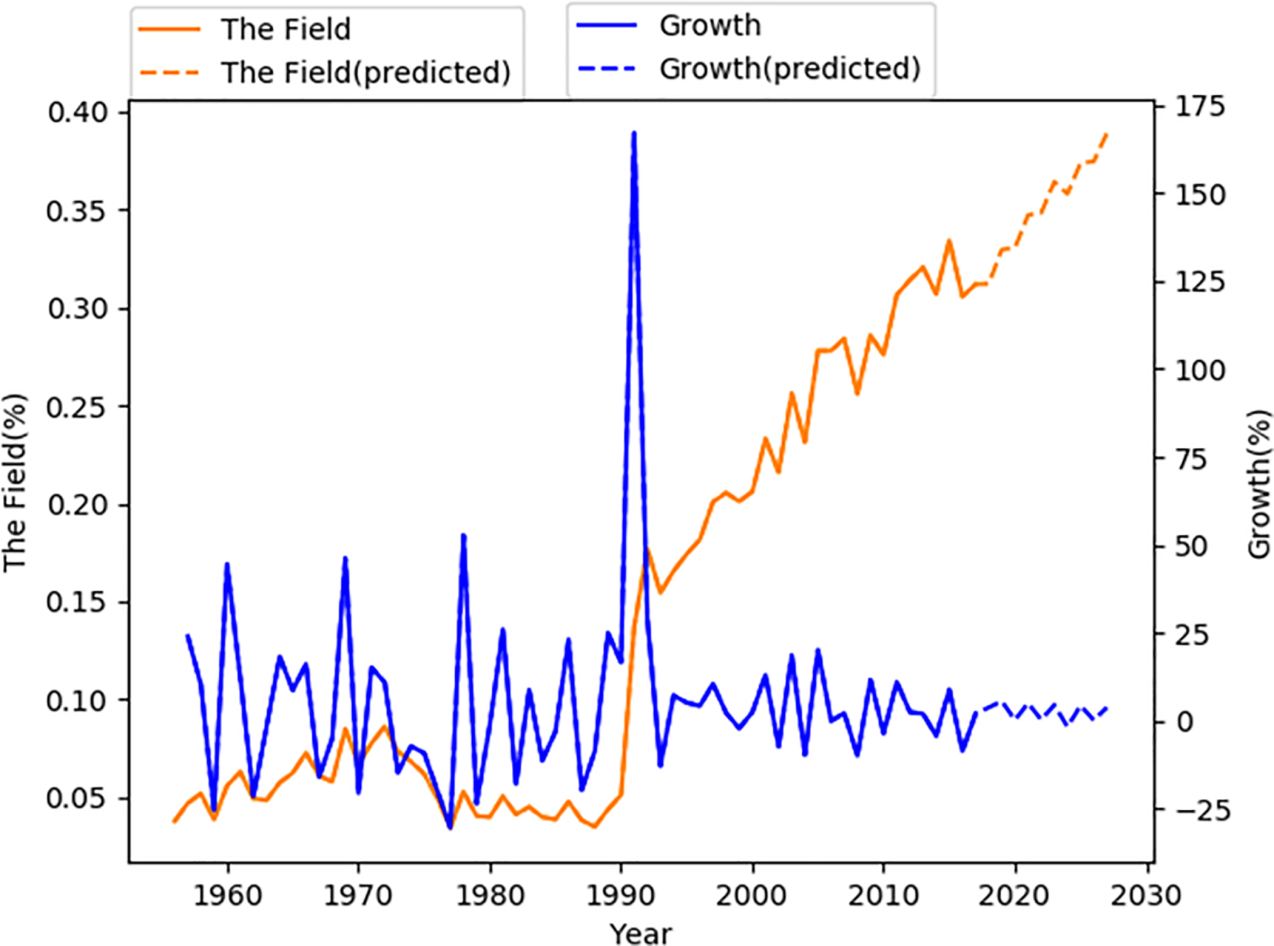
The publication percentage and its growth rate of the field “social class & inequality.

Fig 1 depicts the yearly distribution of articles in terms of annual article counts and the percentage of our sample article counts to the total number of SSCI articles per year (hereafter, publication percentage). The field has grown substantially over the last seven decades. There were only 12 articles (0.04%) published in 1956, but this figure changed to 1,001(0.31%) in 2017. The average annual growth rate in the field reached 5.99%. A systematic change in both series of article count and of publication percentage can be identified over time. The year of 1991 is a change point in the field, as the growth rate in this year jumped from 16.71% in the previous year to 166.98%. And from 1991 onward, the publication percentage (mean = 0.24%, std. = 0.06%) was much higher than that in previous years (mean = 0.05%, std. = 0.02%).

The authors of these articles are from 128 countries, especially USA (36.69%), UK (25.64%) and Canada (5.96%). The ten most frequent organizations in the sample are University College London (2.89%), Harvard University (2.05%), University of Michigan (1.91%), University of Helsinki (1.79%), University of Edinburgh (1.55%), University of Bristol (1.44%), University of Toronto (1.33%), Karolinska Institute (1.29%), University of Cambridge (1.28%), and University of Copenhagen (1.22%).

The articles spread in 112 WoS research areas. Table 2 summarizes Top 10 research areas, which account for around 93.33% of the sample articles. These articles were published in 2,495 journals, among which, *Social Science Medicine*, *Journal of Epidemiology and Community Health*, and *European Journal of Public Health* are the three most frequent outlets in the field (see Table 3).

**Table 2:** Top 10 research areas.

Research areas	Percentage
Public Environmental Occupational Health	24.05
Sociology	18.90
Psychology	14.29
Education Educational Research	9.53
Biomedical Social Sciences	5.75
Social Sciences Other Topics	5.47
Psychiatry	5.27
Business Economics	4.09
Anthropology	3.04
General Internal Medicine	2.94

**Table 3:** Top 10 research outlets.

Source Titles	Percentage
Social Science Medicine	3.42
Journal of Epidemiology and Community Health	2.497
European Journal of Public Health	1.302
Research in Social Stratification and Mobility	1.116
BMC Public Health	1.089
British Journal of Sociology of Education	1.049
American Journal of Public Health	0.996
PLOS One	0.877
International Journal of Epidemiology	0.87
Sociology the Journal of the British Sociological Association	0.863

### Grid search of the optimal number of topics

We first built a corpus containing the titles, keywords, and abstracts of all sample articles. All texts were converted to lower case. We removed stop-words as well as punctuation based on the standard NLTK list and reduced the remaining words to their stems. We then used an algorithm developed by Wang, McCallum, & Wei [20] to replace n-grams with compound words in the text documents. To speed up the modelling process, we followed Blei and Lafferty [13], Hornik and Grun [21], and Antons et al [12] in including only the terms in a topic model whose term-frequency-inverse-document-frequency (tf-idf) values are just above the median of all tf-idf values of the entire vocabulary. These preprocessing procedures resulted in a DTM for further analyses.

**Fig 2:**
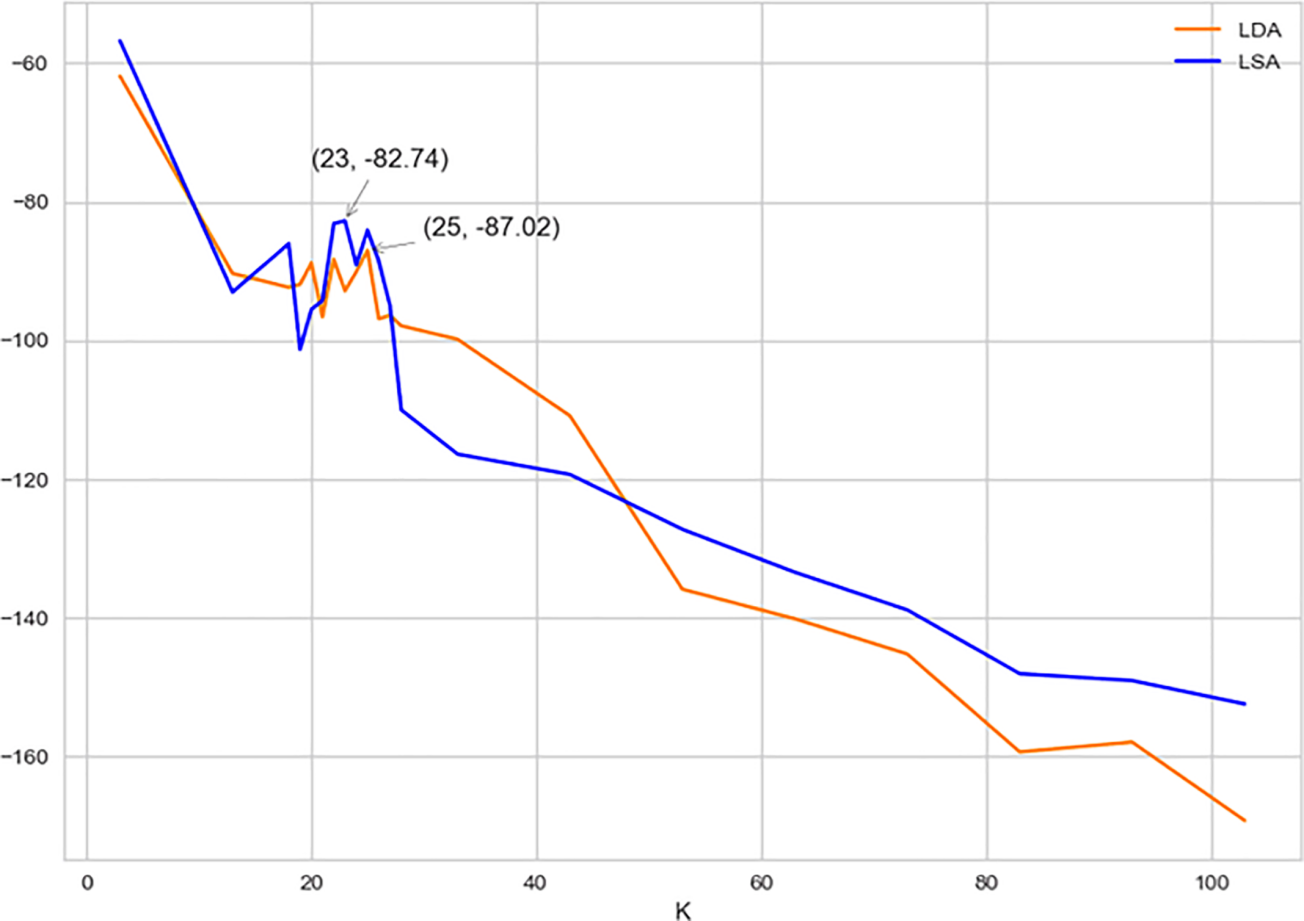
The semantic coherence scores of two-stage grid search for the optimal number of topics.

We conducted LDA topic modeling analysis with the Genism package [22]. The first step was to perform a two-stage grid-search procedure [12] to find the optimal number of topics in our collection. We computed a model set of 3–103 topics in step of 10 (i.e. 3, 13, 23 ∆103), each of which repeats 30 times circumvent the impact of random resampling within LDA. Each model was evaluated by the semantic coherence score with the algorithms of Newman, Lau, Grieser, & Baldwin [23] and of Mimno, Wallach, Talley, Leenders, & McCallum [24]. A good topic model with the optimal number should make the semantic coherence score as large as possible [25]. The first-stage grid search procedure suggested that the semantic coherence score was the largest (-61.91) when number of topics *k* was three and the second largest (-99.81) when *k* was 33. Given that it is unlikely to categorize a large collection of articles like ours into just three topics, we decided the optimal number of topics of the first-stage grid search procedure as *k*_*first-stage*_ = 33. Then we conducted the second-stage grid search procedure by computing a model set of *k*_*first-stage*_ +/- 10 in step of one (i.e. 23, 24, 25,…,42, 43). The second stage procedure suggests that the topic coherence score reaches its maximum when the number of topics is 25. Then, we used Latent Semantic Analysis (LSA) to re-do the two-stage grid-search procedure for the sake of robustness check. The topic coherence scores of LSA were also shown in Fig 2, in which the best topic number seems to be 23 (see Fig 2). These results suggested that our collection of articles could be modelled into more than 20 but less than 30 topics. Note that LDA is proved to be more accurate and robust than LSA [7]. Therefore, we chose the result obtained from the LDA grid-search analysis (25).

**Fig 3:**
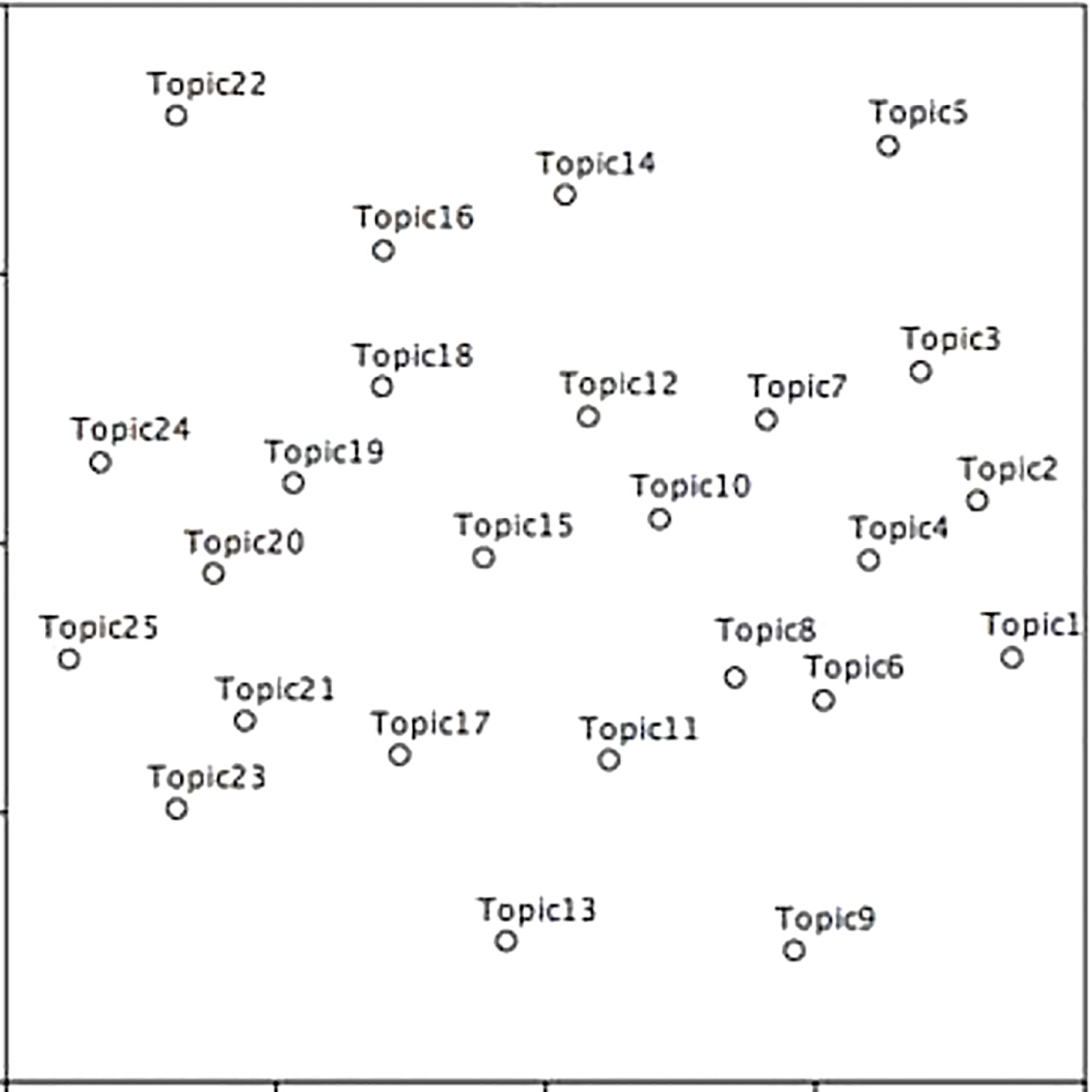
Inter-topic distances in a two-dimensional space.

We assessed topic modeling quality in the following ways. Firstly, we plotted the distances of 25 topics in Fig 3 with the multidimensional scaling (MDS) method. Fig 3 confirms the high quality of the 25-topic model, as topics do not cluster but spread evenly through unit spaces.

Then, we computed the likelihood of each article covering each of the 25 topics with LDA. Note that LDA is a mix-membership model, which means that each document is represented as a mixture of a set of topics and each topic is regarded as a distribution over the words in the vocabulary [26]. We assigned each article to the dominant topic whose topic loading was the highest. We presented the topic modeling results in Table 4. The values of the highest topic loadings of these articles range from 0.96 to 0.11 (mean = 0.56, std. = 0.14). Antons et al [12] argue that an article does not contain a meaningful topic if the loading to this topic is smaller than 0.10. Therefore, the highest topic loadings of all articles were valid.

**Table 4:** Topic modeling results.

Cluster	ID	Topic Labels	#Articles	Loading (σ)
Medicine	1	Drug dependence and disorders among the youth in different social classes	443(3.16%)	0.33(0.79)
Medicine	2	Skeletal, dental and cranial anthropology and social stratification throughout history	346(2.46%)	0.34(0.61)
Social	3	Social class schema and theoretical debates	584(4.16%)	0.27(1.7)
Medicine	4	Preventive health inequality	252(1.80%)	0.32(0.65)
Social	5	Globalization, modernization and social class evolution	1172(8.35%)	0.41(1.49)
Medicine	6	Heart disease, work environment and social inequality	348(2.48%)	0.37(0.57)
Social	7	Discrimination, social value and gender and racial inequality	396(2.82%)	0.29(0.93)
Medicine	8	Cancer and social inequality	359(2.56%)	0.43(0.5)
Social	9	Education and social inequality	1093(7.79%)	0.41(1.48)
Social	10	Criminal justice, terrorism, lifestyle exposure and victimization in different social classes	266(1.89%)	0.32(0.43)
Medicine	11	Cognitive abilities and socioeconomic statues	486(3.46%)	0.4(0.76)
Social	12	Stereotype, ideological orientations and social inequalities	441(3.14%)	0.38(0.91)
Medicine	13	Mortality and social inequality	741(5.28%)	0.44(1.21)
Medicine	14	Community health, intervention and social inequality in multicultural contexts	832(5.93%)	0.34(1.79)
Social	15	Sociolinguistic research and social inequality	301(2.14%)	0.35(0.49)
Social	16	Income inequality, labor market reform and industrial relations	729(5.19%)	0.37(1.38)
Medicine	17	Prenatal care and childhood mental health in different social classes	563(4.01%)	0.34(1.07)
Social	18	Political election and party choices in different social classes	372(2.65%)	0.4(0.62)
Medicine	19	Spatio-temporal inequality, environmental inequality and healthcare	486(3.46%)	0.34(0.92)
Medicine	20	Smoking, diet and active health promotion activities in different social classes	558(3.97%)	0.38(0.75)
Medicine	21	Childhood social class and adulthood health	504(3.59%)	0.37(0.78)
Social	22	Urban inequalities, corporate social responsibility and public policy in connected capitalism	1007(7.17%)	0.44(1.44)
Medicine	23	Oral health and social inequality	659(4.69%)	0.32(1.19)
Medicine	24	Developmental psychology and parents’ child-rearing values and practices	549(3.91%)	0.32(0.94)
Medicine	25	Pathways of social inequalities and psychosocial health	551(3.93%)	0.27(1.6)

Finally, we evaluated the level of topic diversity with the Herfindahl-Hirschman Index (HHI), which has been used in a commonly accepted measure of market or portfolio diversification. As a rule of thumb, a market with an HHI of less than 0.10 is a competitive or diverse marketplace, an HHI of 0.10 to 0.25 is a moderately concentrated marketplace, and an HHI of 0.25 or greater is a highly concentrated or monopolistic marketplace [27]. Analogically, for each article, we squared the topic loading of each topic, and then summing the resulting numbers, which can range from close to zero to one. We followed the same vein of market competition analysis to define that an article contains diverse topics if its HHI is smaller than 0.10; an article contains important topics if its HHI is of 0.10 to 0.18; an article contains a salient topic if its HHI is 0.18 or greater. If there are many articles of diverse topics, then the number of topics chosen may be problematic, as LDA fails to extract dominant topics that are distinct from other topics. We found that 57.71% of the articles are of a salient topic, 38.60 of a few important topics while only 3.69% are of diverse topics. The MDS, the analyses of topic loadings and of topic diversity provide solid supports to the fact that our LDA topic model with 25 topics is of high quality, as the significant topics hidden in each article have been successfully retrieved.

### Topic landscape

We manually labeled each topic in the following manner. Firstly, we downloaded the full texts of the 20 articles whose loadings were the highest within each topic and invited 50 graduate students to read them carefully. That is, each student read 20 randomly-chosen articles and each article was read by two students. Each student proposed a preliminary label for each topic. At the same time, the author team read the abstracts of the 50 highest loading articles per topic. Finally, the author team organized several workshops with the students to finalize the labels. For 21 of the 25 topics, the students suggested labels that were identical or highly similar to those generated by the author team. We discussed the four topics for which the labels assigned by the students and the author team differed significantly to reach a consensus on the most appropriate topic labels.

The number of articles per topic ranges from 252 to 1,172 (mean = 562.2, std. = 249.00). The three most prevalent topics are “globalization, modernization and social class evolution” (Topic 5), “education and social inequality” (Topic 9) and “urban inequality, corporate social responsibility and public policy in connected capitalism” (Topic 22), each of which contains more than 1,000 articles. The three least prevalent topics are “preventive health inequality” (Topic 4), “criminal justice, terrorism, lifestyle exposure and victimization in different social classes” (Topic 10), and “sociolinguistics and social inequality” (Topic 15), each of which contains fewer than or around 300 articles. In addition, “urban inequality, corporate social responsibility and public policy in connected capitalism” (Topics 22), “mortality and social inequality” (Topic 13), and “cancer and social inequality” (Topic 8) exhibit the three highest average loadings (>0.42), indicating that the articles covering these topics tend to be more similar than those covering relatively low-loading ones, for example, “social class schema and theoretical debates” (Topic 3, average loading = 0.26), “discrimination, social value, and gender and racial inequality” (Topic 7, average loading = 0.29) and “pathways of social inequality and psychosocial health” (Topic 25, average loading = 0.28).

Finally, we listed the three most cited references and the three most frequent outlets per topic in Tables 5 and 6. These cited references and outlets can be regarded as the field’s principal knowledge sources. In general, Krieger, Williams, & Moss [28] has been cited in 12 topics, and Liberatos, Link, & Kelsey [29] in nine. Pierre Bourdieu’s work [30,31] is also extensively and widely cited in many topics. In addition, *Social Science & Medicine* is one of Top 3 outlets in 16 topics, *Journal of Epidemiology and Community Health* in 10 topics, and *American Journal of Public Health* in five topics.

**Table 5:** The three most cited references per topic.

1	Muntaner, Eaton, Diala, Kessler & Sorlie [54]; Krieger, Williams, & Moss [28]; Hollingshead [55].
2	Ambrose [56]; Phenice [57]; Hayden [58].
3	Goldthorpe [59]; Stanworth [60]; Dahrendorf [61].
4	Marmot & Smith [62]; Davis [63]; Smaje & Le Grand [64].
5	Reay [65]; Peterson & Kern [66]; Bourdieu [31]
6	Rosengren, Wedel, & Wilhelmsen [67]; Marmot, Rose, Shipley, & Hamilton [68]; Karasek, [69].
7	Kessler, Mickelson, & Williams [70]; Karlsen & Nazroo [71]; Williams, Neighbors, & Jackson [72].
8	Farley & Flannery [73]; Krieger et al. [74]; Clegg et al. [75].
9	Raftery & Hout [76]; Erikson & Goldthorpe [77]; Mare [78].
10	Steensland et al [79]; Wright, Caspi, Moffitt, Miech, & Silva [80]; Hindelang, Hirschi, & Weis [81].
11	Whalley & Deary [82]; Hollingshead & Redlich [83]; Brayne & Calloway [84].
12	Kraus & Keltner [85]; Pratto, Sidanius, Stallworth, & Malle [86]; Tajfel & Turner [87].
13	Huisman et al. [88]; Marmot & Mcdowall [89]; Kunst, Groenhof, Mackenbach, & Hlth [90].
14	Bronfenbrenner [91]; Liu, Soleck, Hopps, Dunston, & Pickett [92]; Adler, Epel, Castellazzo, & Ickovics [93].
15	American Psychiatric Association [94]; Trudgill [95]; Labov [96].
16	Erikson, Goldthorpe, & Portocarero [97]; Sorenson [98]; Shavit & Blossfeld [99].
17	Brooke, Anderson, Bland, Peacock, & Stewart [100]; Pattenden, Dolk, & Vrijheid [101]; Lynch [102].
18	Evans [103]; Inglehart [104]; Hout, Brooks, & Manza [105].
19	Smith, Hart, Watt, Hole, & Hawthorne [106]; OCampo, Xue, Wang, & Caughy [107]; Liberatos et al. [29].
20	Liberatos et al. [29]; Galobardes, Shaw, Lawlor, Lynch, & Smith [108]; Marshall et al. [109]
21	Lynch, Kaplan, & Salonen [110]; Krieger et al., [28]; Poulton et al. [111].
22	Krieger, Okamoto, & Selby [112]; Harvey [113]; Bian [114].
23	Townsend & Nick [115]; Ware & Sherbourne [116]; Adler et al. [117].
24	Bourdieu & Passeron [30]; Burkam, Ready, Lee, & LoGerfo [118]; Bourdieu [31]
25	Wilkinson [119]; Kitagawa & Hauser [120]; Radloff [121].

**Table 6:** The three most popular outlets per topic.

1	Social Psychiatry and Psychiatric Epidemiology; Psychological Medicine; British Journal of Psychiatry
2	American Journal of Physical Anthropology; Journal of Archaeological Science; Journal of Anthropological Archaeology
3	Sociology; British Journal of Sociology; Social Science & Medicine
4	Social Science & Medicine; European Journal of Public Health; BMC Health Services Research
5	Sociology; British Journal of Sociology of Education; Sociological Research Online
6	Social Science & Medicine; Journal of Epidemiology and Community Health; American Journal of Epidemiology
7	Social Science & Medicine; American Journal of Public Health; Sex Roles
8	Cancer Causes & Control; American Journal of Public Health; Journal of Epidemiology and Community Health
9	British Journal of Sociology of Education; Research in Social Stratification And Mobility; Sociology Of Education
10	Review of Religious Research; Journal for the Scientific Study of Religion; Criminology
11	Intelligence; Personality and Individual Differences; International Journal of Geriatric Psychiatry
12	Journal of Personality and Social Psychology; Personality and Social Psychology Bulletin; Journal of Social Issues
13	Journal of Epidemiology and Community Health; Social Science & Medicine; Scandinavian Journal of Public Health
14	Social Science & Medicine; Teaching Sociology; Sociology of Health & Illness
15	Journal of Sociolinguistics; British Journal of Psychiatry; Language in Society
16	Research in Social Stratification and Mobility; European Sociological Review; Social Science & Medicine
17	Journal of Epidemiology and Community Health; European Journal of Public Health; American Journal of Epidemiology
18	Electoral Studies; British Journal of Political Science; European Sociological Review
19	Social Science & Medicine; Journal of Epidemiology and Community Health; Health & Place
20	BMC Public Health; Journal of Epidemiology and Community Health; Preventive Medicine
21	Journal of Epidemiology And Community Health; Social Science & Medicine; International Journal of Obesity
22	Social Science & Medicine; Urban Studies; Environment and Planning A
23	Social Science & Medicine; Journal of Epidemiology and Community Health; European Journal of Public Health
24	Sociology of Education; Social Science Research; Social Science & Medicine
25	Social Science & Medicine; Journal of Epidemiology and Community Health; Journal of Health and Social Behavior

### Topic dynamics

Given that the field in general has experienced substantial growth after 1991, we discussed the temporal dynamics of each topic in two periods (i.e. 1956–1990 and 1991–2017). We constructed 26 time series (i.e. the field and the 25 topics, shown in Fig 1 and S1 Fig). The publication percentage of the field has grown significantly in both pre-1991 (mean = 3.03%) and post 1991 periods (mean = 9.12%). There are 16 topics that experienced a decline before 1991 but all of them strongly bounded up after 1991. For example, the publication percentage of “Cancer and social inequality” (Topic 8) shrink (on average -26.11% per year) before 1991 but expanded (on average 6.71% per year) in the second period. None of the 25 topics declined in the post-1991 period. In particular, “smoking, diet and active health promotion activities in different social classes” (Topic 20) has increased on average 54.94% per year, “heart disease, work environment and social inequality” (Topic 6) increased on average 39.61% and “education and social inequality” (Topic 9) increased on average 26.05%.

Some topics, such as “smoking, diet and active health promotion activities in different social classes” (Topic 20), “childhood social class and adulthood health” (Topic 21), and “preventive health inequality” (Topic 4), did not appear in the 1950s and 1960s. It was not until the 1990s that all 25 topics were present. “Social class schema and theoretical debates” (Topic 3) was prevalent in 1960s and 1970s but suddenly becomes much less popular in the following decades.

Then, we intended to identify the trends in the filed as a whole and in each topic using time series forecasting technique. We did not follow conventional trend analysis to employ linear and quadratic time trend regressions for the series of article counts. That is because, on the one hand, article count series usually exhibits strong autocorrelation, which manifests in correlated residuals after a regression model has been fit. The autocorrelation violates the standard assumption of independent errors [32]. On the other hand, article counts do not take the consistent growth in all SSCI publications over time into account, which makes the results obtained by regressions spurious. Therefore, we chose Autoregressive Integrated Moving Average (ARIMA) technique. The AR part can be conceived as a linear regression on previous time series values and the MA part is conceptually regarded as a linear regression of the current value of the series against prior random shocks. The I (for “integrated”) part the data values have been replaced with the difference between their values and one or several previous values, which allow non-stationary series to be modeled. Explicitly catering to a suite of standard structures in time series data, ARIMA provides a simple yet powerful method for making skillful time series forecasts [33].

We constructed 26 time series and identified the appropriate ARIMA terms following the conventional Box-Jenkins Methodology [33]:

Firstly, we split a series into a training part (80%, i.e. 1956–2005) and a test part (20%, i.e. 2006–2017). We used the Augmented Dickey–Fuller test to identify the appropriate order of differencing (i.e. the *d* parameter) for the training series. Secondly, we specified the number of AR order with the partial autocorrelation function (PACF) plot for the training series. The PACF displays the autocorrelation of each lag of a series after controlling for the auto correlation caused by all preceding lags [34]. If there is a sharp drop in the PACF of a series after *p* lags, then an ARIMA model should include *p* autoregressive terms as the previous *p*-values are responsible for the autocorrelation in the series [35]. Thirdly, we specified the number of MA terms by plotting the ACF of the training series. If the ACF is non-zero for the first *q* lags and then drops toward zero, then an ARIMA model should include *q* MA terms [34]. Fourthly, we fitted an ARIMA with the identified order parameters (i.e. *p*, *d*, *q*) to the training series. To verify the quality of this model, we plotted its residual to see whether it appears as entirely random white noise and conducted the Ljung-Box test to formally check whether the errors are uncorrelated across many lags [36,37]. Otherwise, we improved the model upon by removing all the remaining trend. Finally, we tested the improved model with the test series and computed the scores of RMSE, AIC and BIC.

To check the robustness of our ARIMA order specifications, we conducted a grid-search by estimating 1,125 ARIMA models with different combinations of orders (i.e. *d* = [0,5], *p* = [0,15], *q* = [0,15]). By comparing these models with the manually specified optimal model in terms of the Ljung-Box test of residuals, AIC and BIC, the ARIMA grid-search results confirm that our order specifications were indeed optimal (i.e. the Ljung-Box test is statistically insignificant and the values of RMSE, AIC and BIC are minimum). Results were summarized in Table 7 and S1 Fig.

**Table 7:** The results of ARIMA and forecasting.

Topic	Order^1^	Log Lik.	AIC	BIC	HQIC	Ljung-Box^2^	Pre-1991 Gth	Post-1991 Gth	Avg Future Gth	Category
The Field	(1, 1, 1)	344.23	-680.47	-672.98	-677.640	0.11(0.74)	3.03%	9.71%	2.51%	Benchmark
Topic 1	(1, 0, 1)	441.95	-875.89	-868.33	-873.023	0.002(0.96)	-22.19%	15.25%	-3.64%	Cold
Topic 2	(0, 0, 6)	465.19	-914.38	-899.25	-908.641	0.38(0.54)	14.67%	12.91%	-241.18%	Cold
Topic 3	(3, 1, 1)	387.20	-762.41	-751.18	-758.167	0.003(0.95)	23.58%	12.19%	-1.42%	Cold
Topic 4	(2, 0, 0)	481.49	-954.99	-947.42	-952.116	0.006(0.94)	-5.75%	4.62%	-6.50%	Cold
Topic 5	(4, 1, 0)	455.30	-898.60	-887.37	-894.357	0.008(0.93)	-19.13%	28.49%	-0.11%	Cold
Topic 6	(4, 0, 0)	464.02	-916.04	-904.69	-911.734	0.086(0.77)	-3.35%	39.61%	0.13%	Stable
Topic 7	(3, 0, 0)	458.57	-907.15	-897.69	-903.561	0.031(0.86)	-19.67%	18.89%	-4.85%	Cold
Topic 8	(3, 0, 0)	474.18	-938.37	-928.91	-934.777	0.097(0.76)	-26.11%	6.70%	-1.61%	Cold
Topic 9	(9, 1, 0)	433.99	-845.98	-825.39	-838.200	0.011(0.91)	17.91%	36.31%	3.69%	Hot
Topic 11	(1, 0, 1)	451.47	-894.95	-887.38	-892.078	0.43(0.51)	-5.24%	10.07%	-2.34%	Cold
Topic 10	(1, 0, 1)	477.77	-947.54	-939.98	-944.673	0.10(0.76)	-3.11%	7.84%	-2.58%	Cold
Topic 12	(0, 1, 1)	444.82	-883.64	-878.03	-881.517	1.49(0.23)	25.25%	25.17%	0.00%	Stable
Topic 13	(6, 1, 3)	446.49	-870.98	-850.39	-863.198	0.40(0.53)	-11.91%	19.68%	-0.32%	Cold
Topic 14	(8, 0, 0)	458.75	-897.49	-878.57	-890.313	0.007(0.93)	-1.76%	11.88%	3.54%	Hot
Topic 15	(1, 0, 0)	455.28	-904.56	-898.89	-902.408	0.06(0.80)	4.84%	14.87%	-20.01%	Cold
Topic 16	(3, 1, 0)	451.93	-893.85	-884.50	-890.316	0.002(0.97)	2.53%	27.63%	1.63%	Stable
Topic 17	(4, 1, 0)	462.62	-913.24	-902.02	-909.000	0.0005(0.98)	-5.86%	20.68%	0.18%	Stable
Topic 18	(0, 1, 1)	448.38	-890.76	-885.14	-888.635	2.90(0.09)	-8.05%	21.97%	0.00%	Stable
Topic 19	(1, 1, 1)	452.23	-896.47	-888.99	-893.641	0.04(0.84)	3.34%	11.71%	0.25%	Stable
Topic 20	(9, 1, 0)	456.72	-891.44	-870.85	-883.658	0.01(0.93)	-18.53%	54.94%	0.37%	Stable
Topic 21	(2, 1, 0)	466.03	-924.06	-916.58	-921.232	0.65(0.42)	-13.63%	24.26%	-0.55%	Cold
Topic 22	(3, 0, 0)	455.68	-901.36	-891.90	-897.769	0.02(0.90)	-7.24%	10.51%	8.53%	Hot
Topic 23	(5, 1, 0)	455.69	-897.37	-884.27	-892.423	0.35(0.55)	-17.13%	28.30%	-2.70%	Cold
Topic 24	(0, 1, 1)	437.37	-868.75	-863.13	-866.625	0.72(0.40)	25.75%	21.49%	0.00%	Stable
Topic 25	(1, 0, 1)	434.51	-861.02	-853.45	-858.148	0.08(0.77)	7.07%	16.60%	-1.30%	Cold

1: the order is listed as *p*, *d*, *q*.

2: the number in parentheses is p-value of the Ljung-Box test.

We employed the optimized ARIMA models to forecast the publication percentages of the field and of each topic for the next ten years (i.e. 2018–2027) respectively. The forecast average annual growth rate was used as the indicator of future topic prevalence (see Table 7). The field may continue to expand in the next decade, as its annual growth rate will be 2.51%, suggesting that the field of social class and inequality will consistently attract significant attention in multidisciplinary research communities. We classified the 25 topics into three categories using the following criteria: *hot* topics for those whose forecast annual growth rates are higher than or equal to the one of the field (i.e. 2.51%), *stable* topics for those whose rates are positive or equal to zero but smaller than the one of the field, and *cold* topics for those whose rates are negative. There are three hot topics, eight stable topics and 14 cold topics. We discussed these findings in the next section.

## Discussion and conclusions

The aim of this study is to provide a systematic review of social class and inequality research over the last seven decades: its evolution, topic landscape, and dynamics. Our topic modelling analyses considerably enhance understanding of the hidden structure of 25 distinct topics covering the overall development in the field. In addition, our analysis of topic dynamics reveals the highly fluctuated nature of the field’s content structure. Our forecasting results suggest that while in general, the field will continue to attract more attention, 14 topics may lose their popularities. In particular, “skeletal, dental and cranial anthropology and social stratification throughout history” (Topic 2) will dramatically shrink -241.18%, followed by “sociolinguistic research and social inequality (Topic 15, -20.01%) and “preventive health inequality” (Topic 4, -6.50%). These findings seem to be reasonable, given that the three topics are not mainstream in the field, all of which took up less than 2.5% of the articles respectively.

In addition, the 25 topics can be roughly divided into two categories. The 15 medicine-related research topics dominate the field, comprising 54.86% of the articles. This is not surprising, given that healthcare, the sociology of illness, and the social organization of medicine are among the fastest growing areas of modern research. Studies in these topics use core principles and concepts of medical sociology to elucidate the determinants and consequences of various types of illness and wellness (e.g. oral health, prenatal care and psychology). These articles have extensively examined the socioeconomic risk factors of health and their iatrogenic repercussions. Such research contributes to the field of social class and inequality by exploring the social meaning of illness, by examining the issue of care-taking as well as care-giving actions related to familial, community and governmental responsibilities, and by deconstructing health inequalities grounded in social stratifications. Our research suggests that in general, the research in these topics has substantially grown and matured, because that the forecast annual growth rates of many medicine-related research topics are either negative or close to zero. That is probably because many studies have reached a consensus that the problems of access to health care, inequality in medical coverage, and the influence of oppressive social structures make ‘health’ impossible for many people confined in an unfavorable class position [38]. Future efforts may be devoted to “community health, intervention and social inequality in multicultural contexts” (Topic 14), whose forecast annual growth rate will reach 8.53%.

The second category of work in our collection is social sciences-oriented, focusing on topics related to education inequality, social structure evolution, the impact of globalization, business development and public policies. There may be research gaps in “education and social inequality” (Topic 9, whose forecast annual growth rate will be 3.69%) and “income inequality, labor market reform and industrial relations” (Topic 16, whose forecast annual growth rate will be 1.63%). Growing inequality is regarded as one of the most important developments in today’s industrial relations. This phenomenon has been most pronounced in the West, where rising support for populism has disrupted politics and challenged corporate capitalism in many countries [39]. Future research may give special attention to emerging forms of organizational restructuring and labor market institutions, such as trade union power, wage regulations and the influence of the Artificial Intelligence-based fourth industrial revolution.

In conclusion, this study applies LDA topic modelling to structure a large text corpus effectively. By doing so, we enable researchers to examine the detailed profile of each topic and estimate its relative salience. By describing the whole body of knowledge at a relatively granular level, we contribute to a rich understanding of the field’s topic landscape. As such, researchers can appreciate the full range of topics and select those they wish to examine in depth. In addition, our topic landscape informs social class and inequality teaching and course design. Instructors can identify important topics to cover in a course, and include relevant articles associated with each topic. Our study also helps postgraduate students and junior researchers identify which research topics to examine. Finally, our findings have many meaningful implications for journal editors. They can compare the field’s current topic landscape against their journal’s editorial priorities, and thus choose promising topics to be reflected in the composition of the editorial board or promoted through special issues.

However, our study may be of some limitations. Our sample articles were collected from WoS. Although it is probably the single most authoritative source for “high-impact” publications and has a relatively better coverage of social sciences and arts/humanities than other academic databases, WoS focuses mainly mainstream journals and articles, especially those in English. As a result, our analyses excluded articles published in emerging journals, in non-English languages and other types of publications (e.g. books, conference papers, technical reports, theses and dissertations). Future studies may collect publication records from Google Scholar, as it covers book contents along with other freely-accessible online publications. In addition, we did not take the correlations between topics into account so that we cannot forecast how the values of one topic will be correlated with those of other topics. Future work may employ multivariate time series methods to capture the associations between topic time series. Finally, we did not specify forecasting models with any external bibliometric factors that may correlate with the growth or decline of a topic time series. Future work should investigate bibliometric determinants of topic dynamics.

## Supporting information

S1 Fig The temporal trajectories of 25 topics. (PDF)
