# Supplementary figures and images for: The research topic landscape in the literature of social class and inequality
Source: PLoS One. 2018 Jul 2;13(7):e0199510. doi: 10.1371/journal.pone.0199510 (PMC6028105; doi:10.1371/journal.pone.0199510)

**S1 Fig: The Temporal Trajectories of 25 Topics**

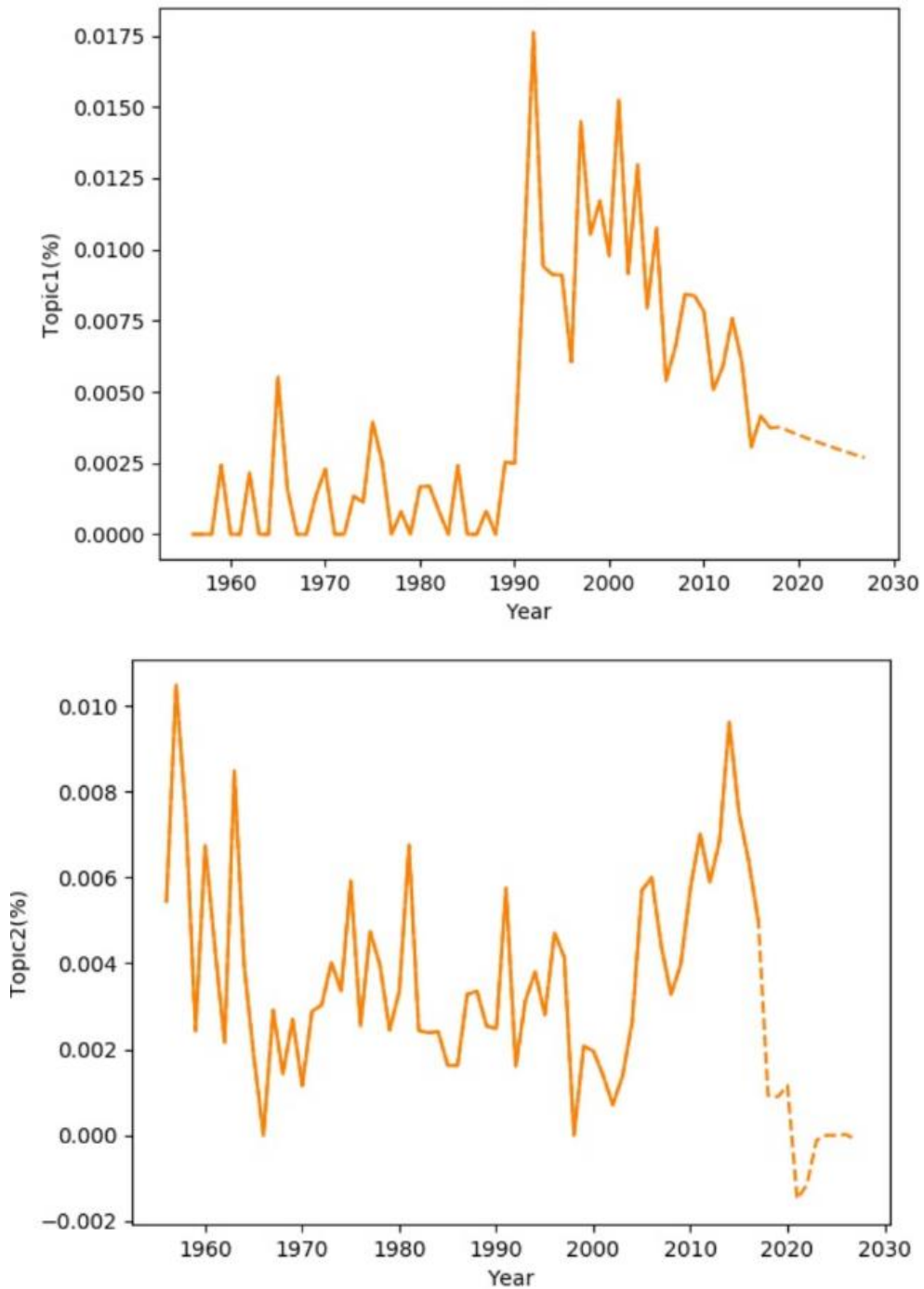

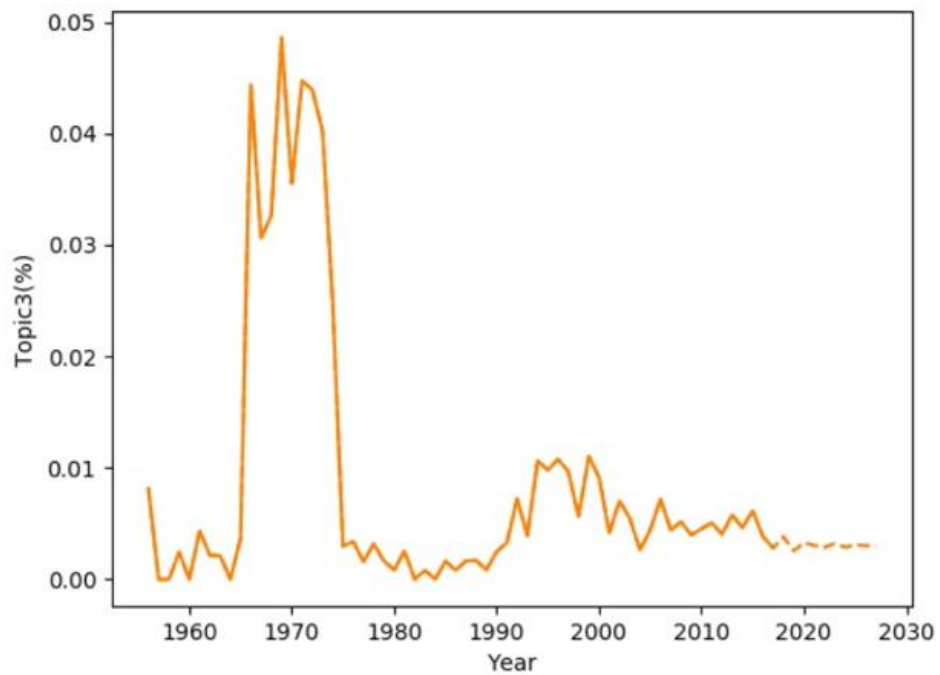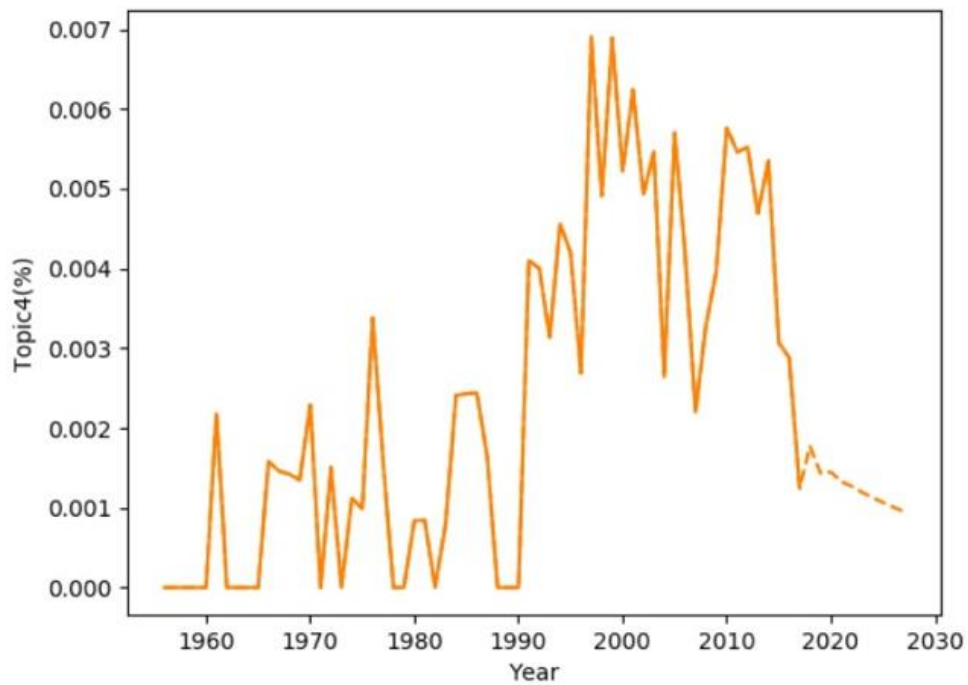

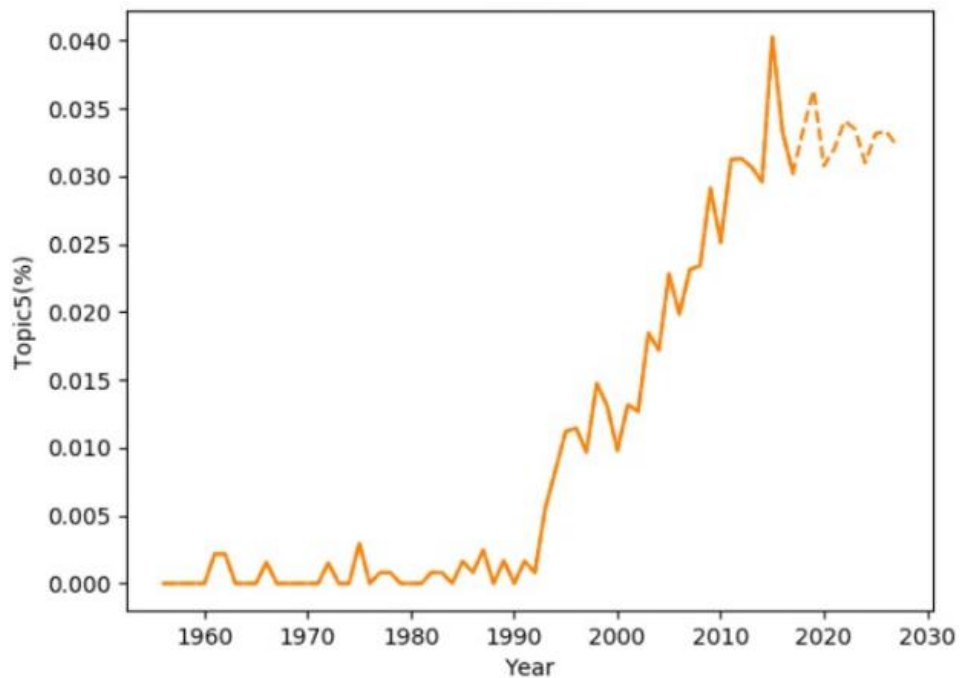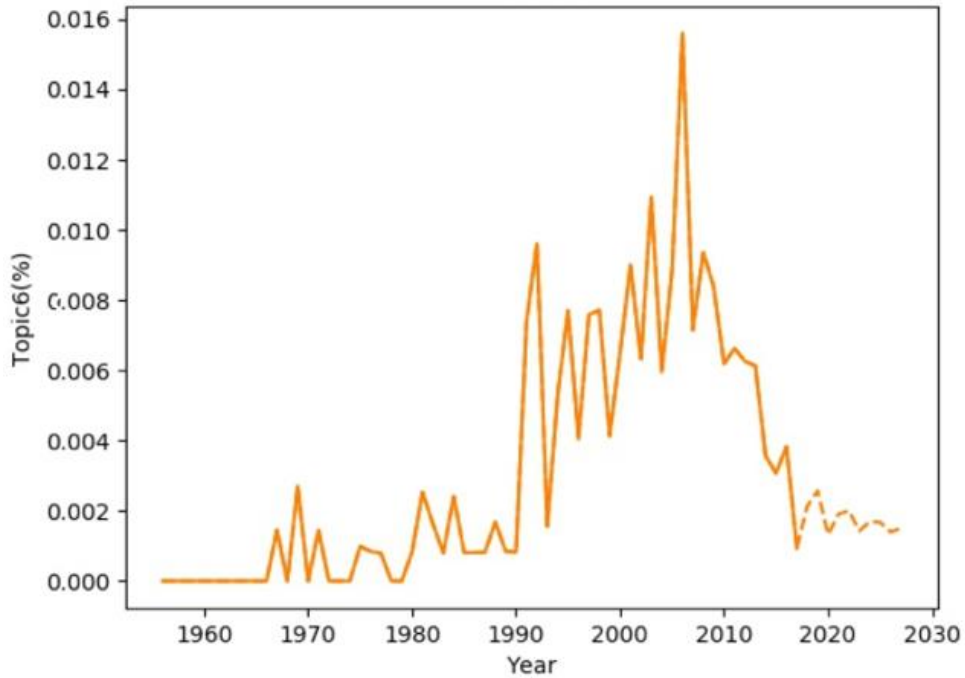

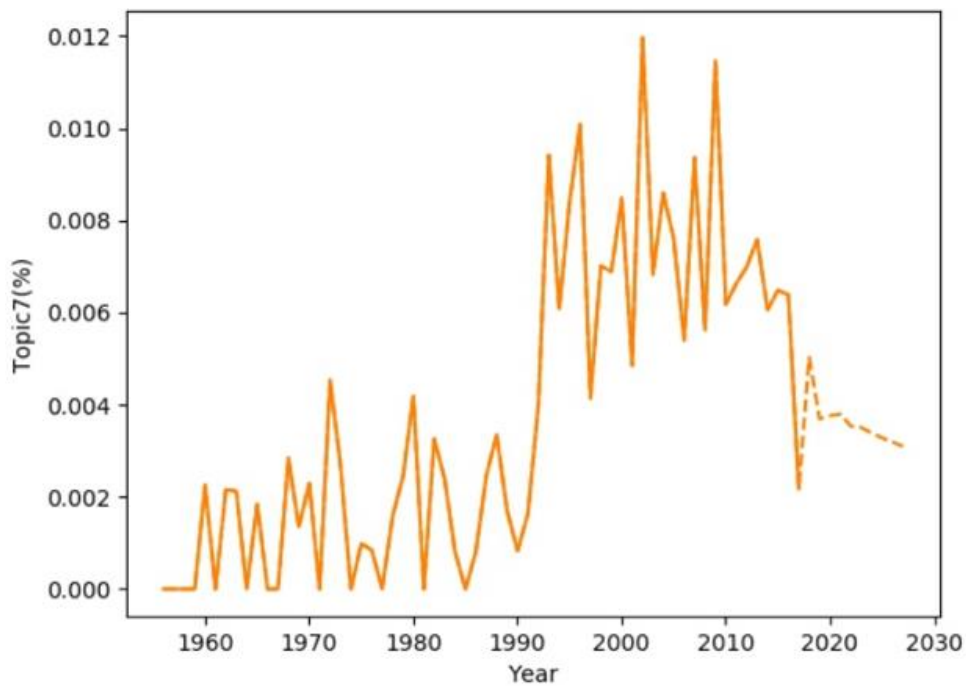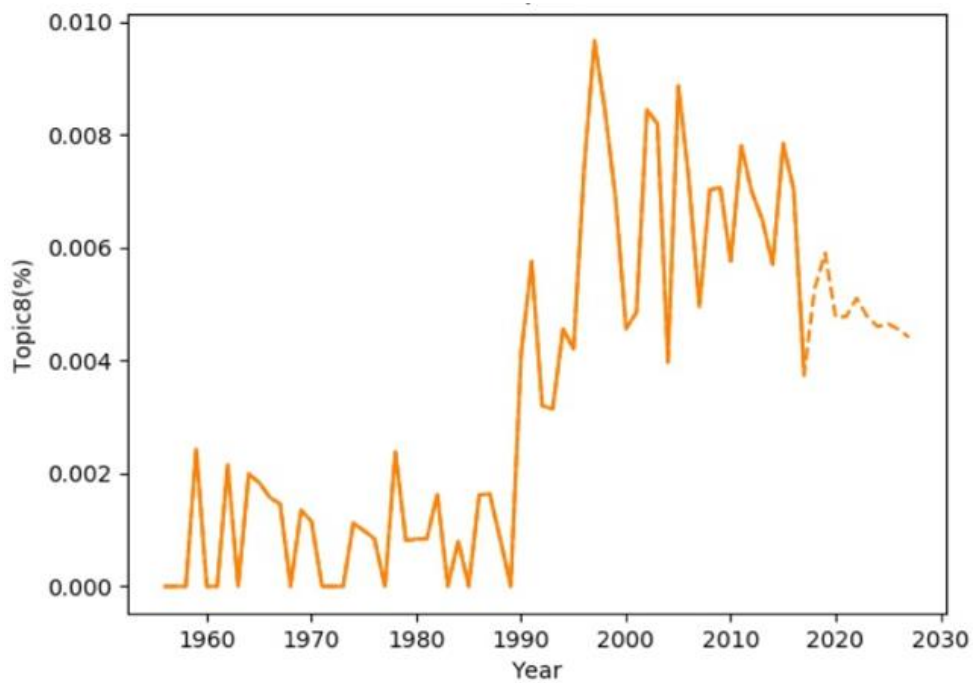

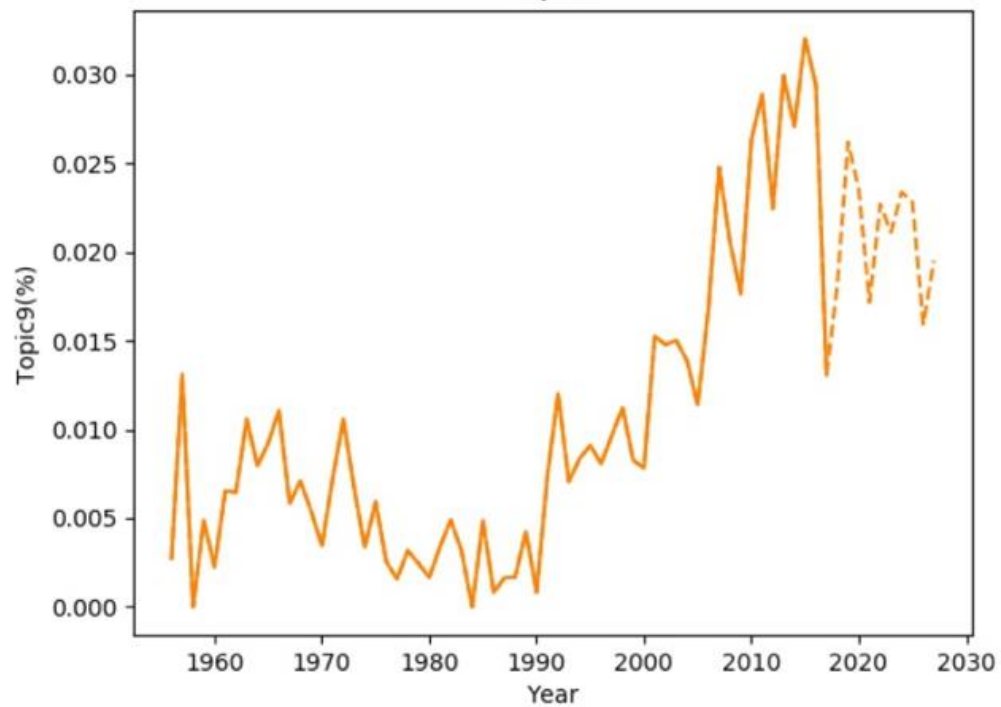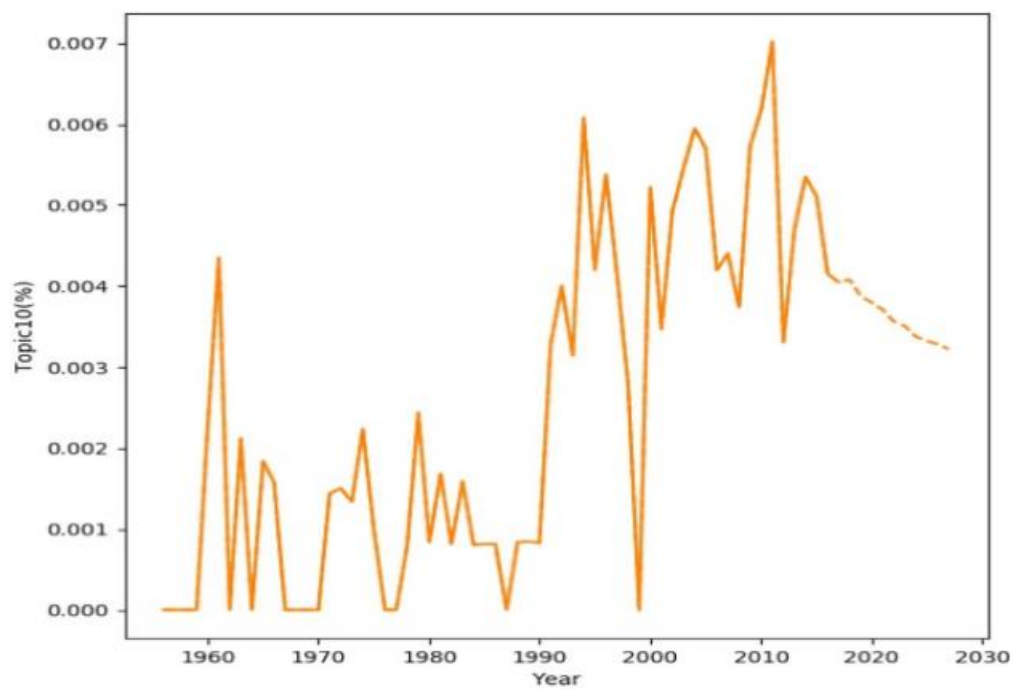

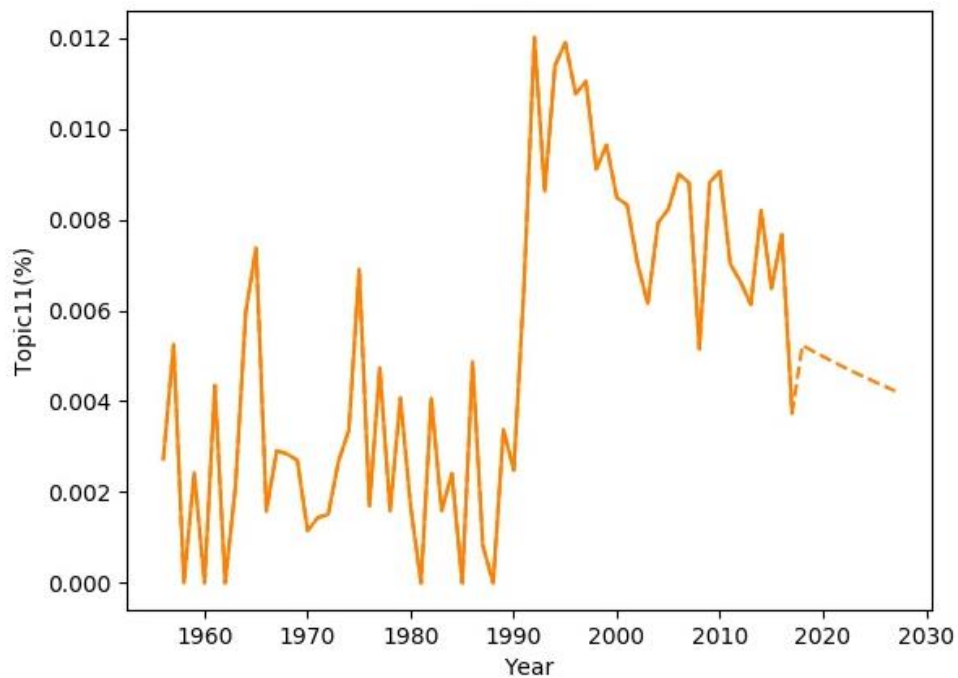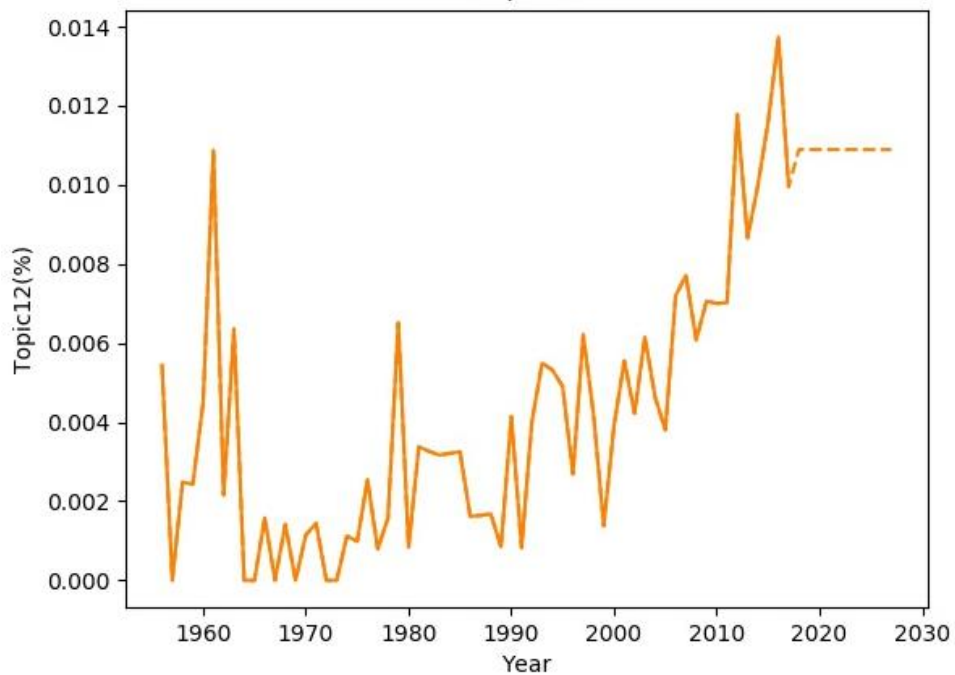

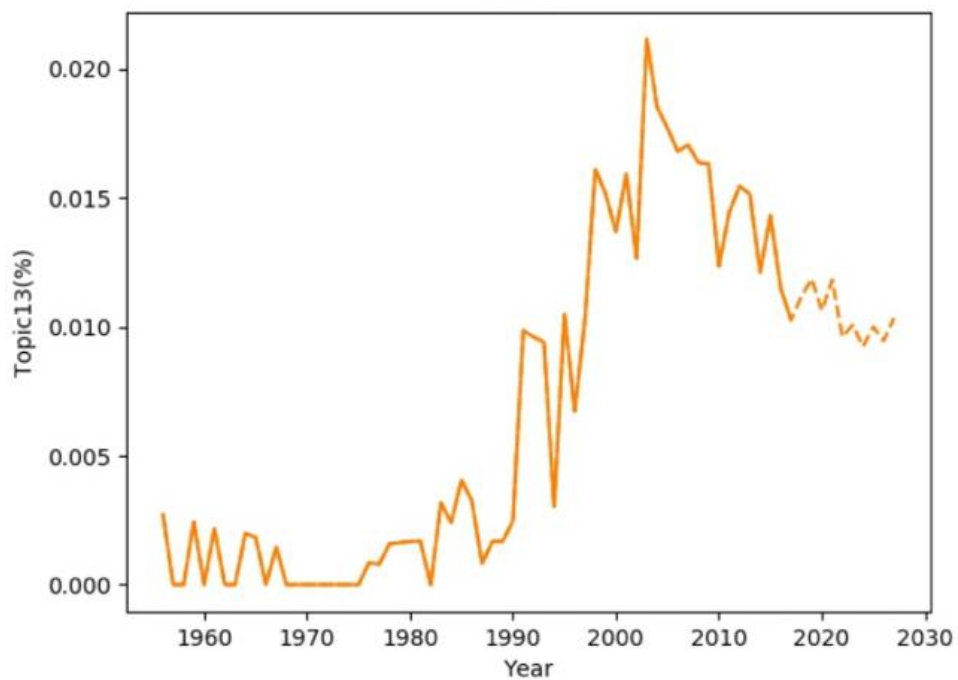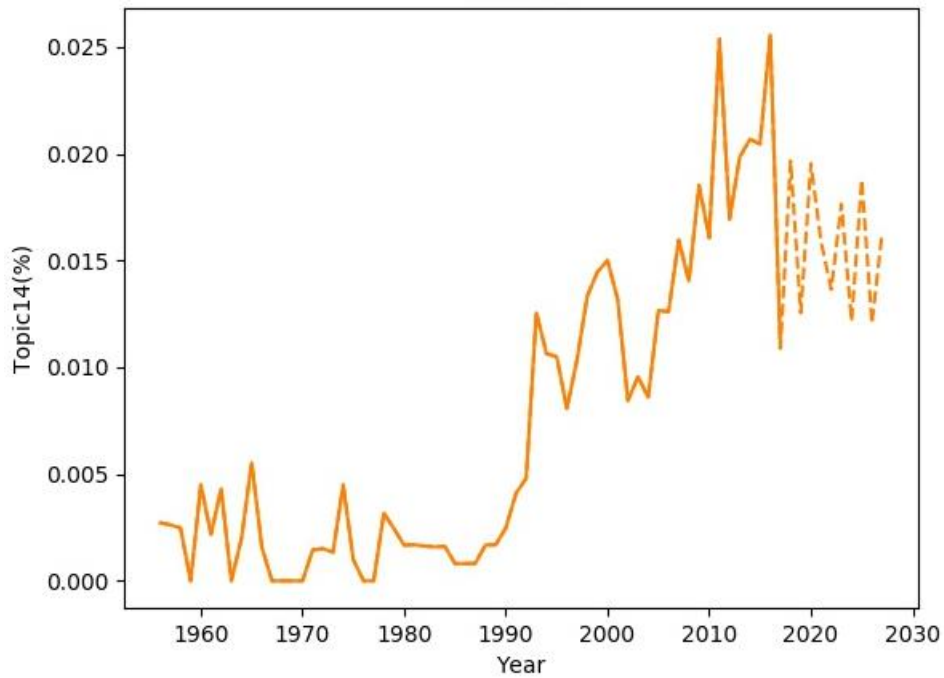

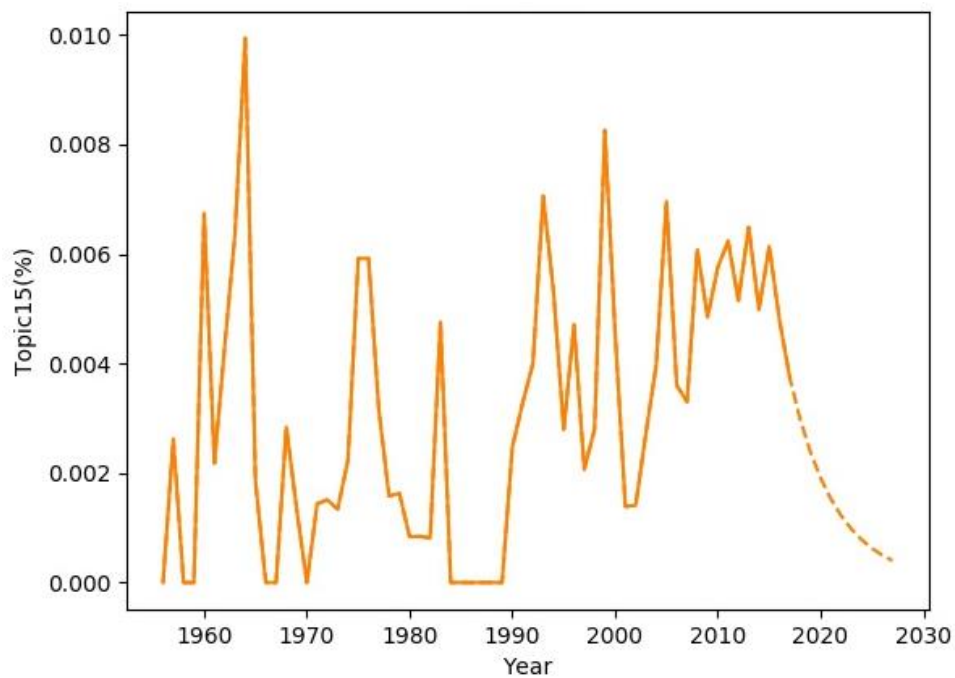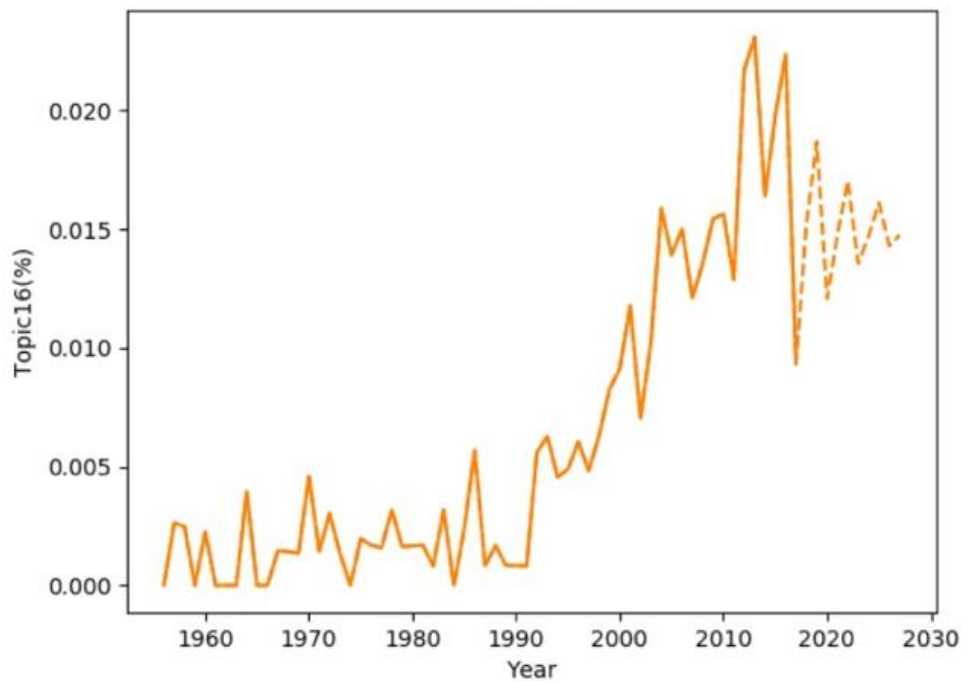

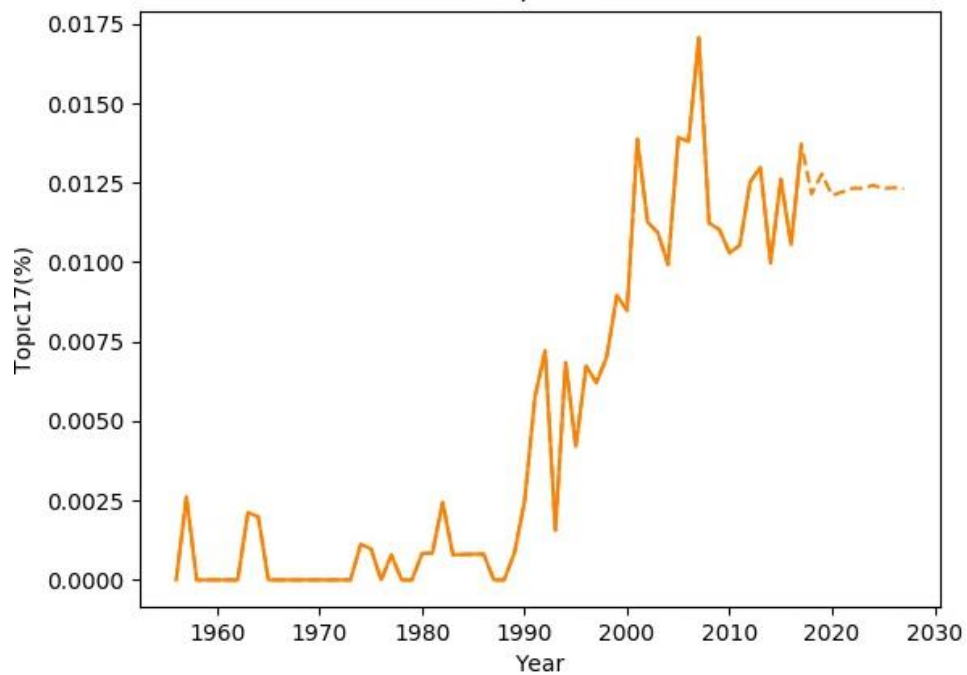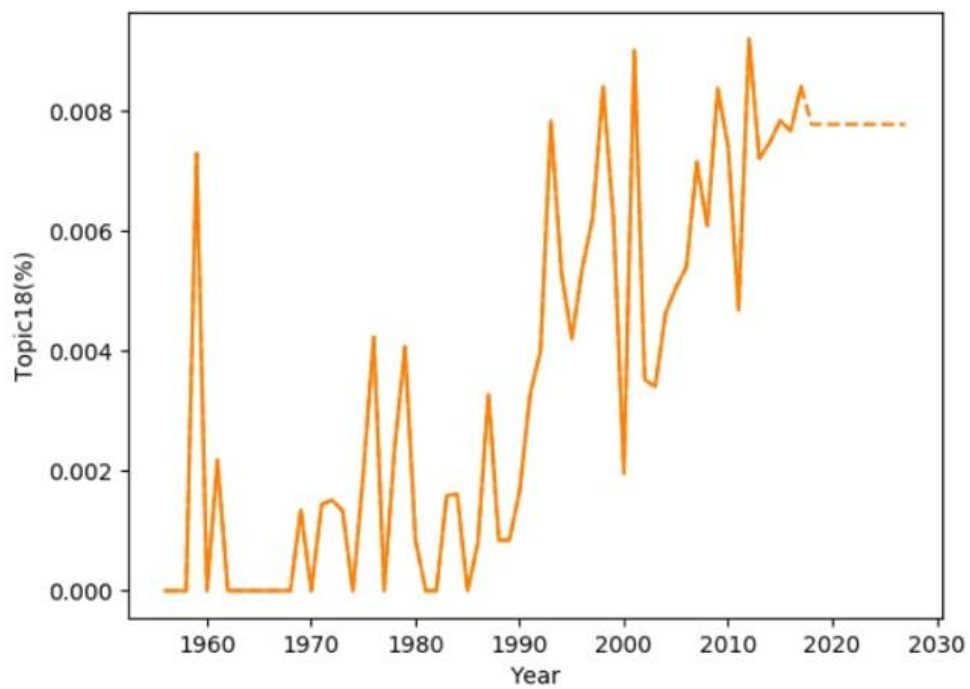

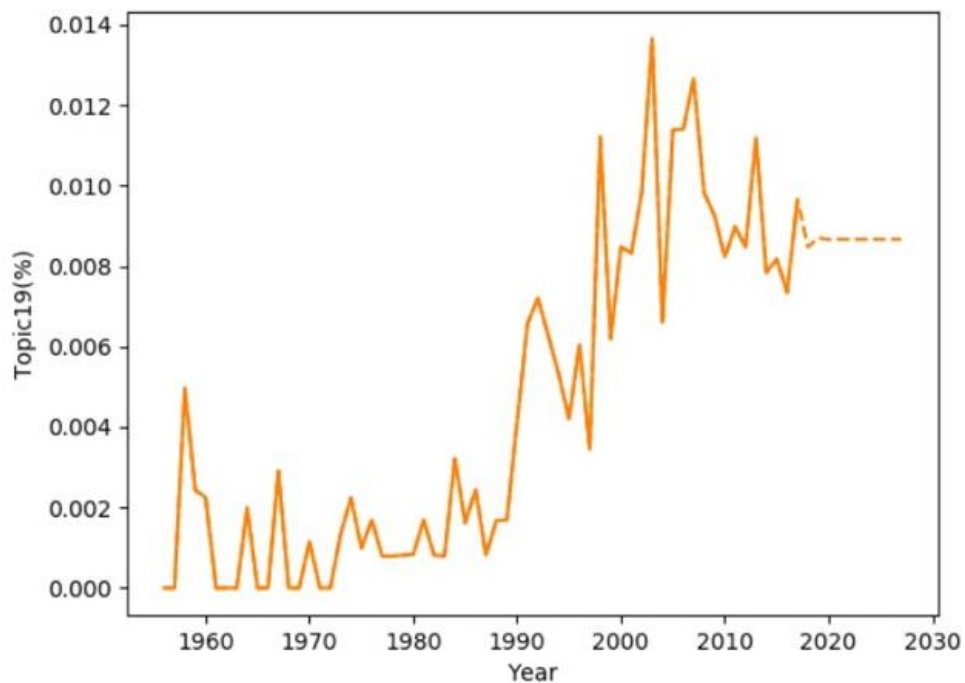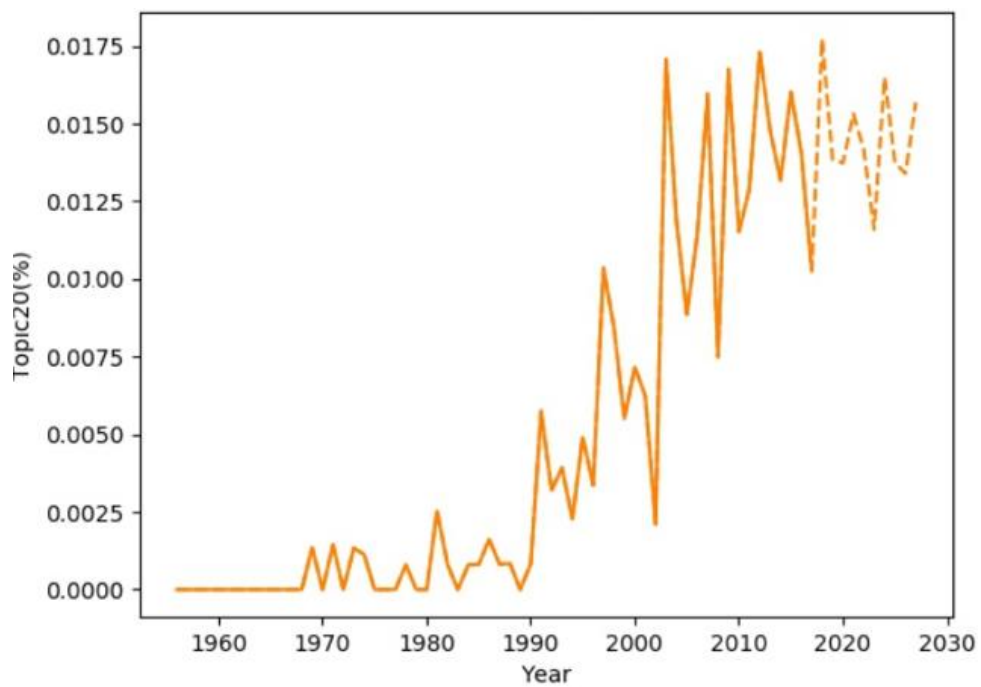

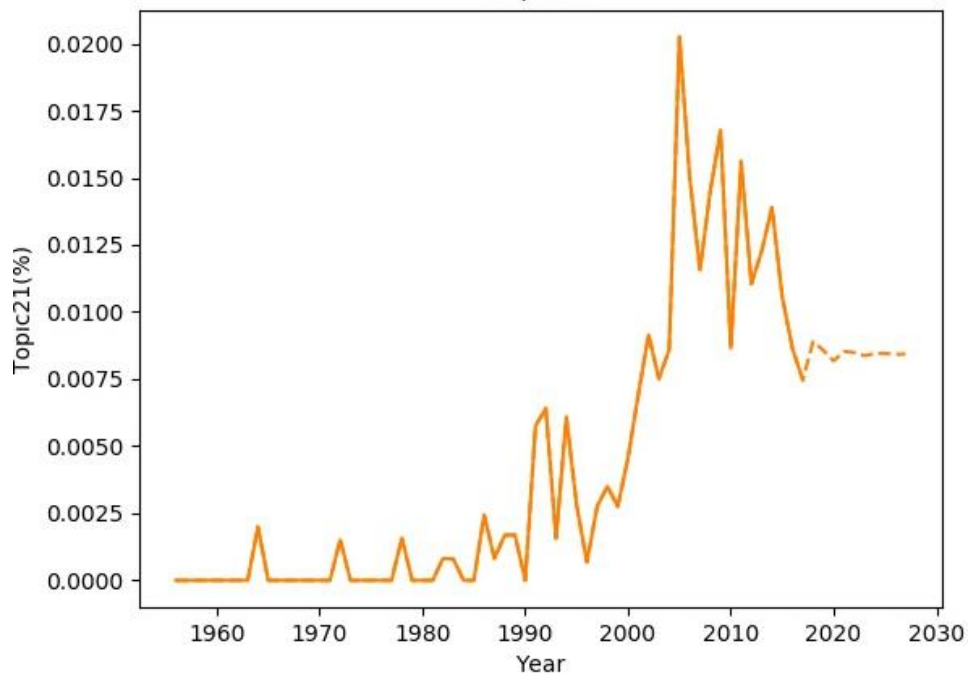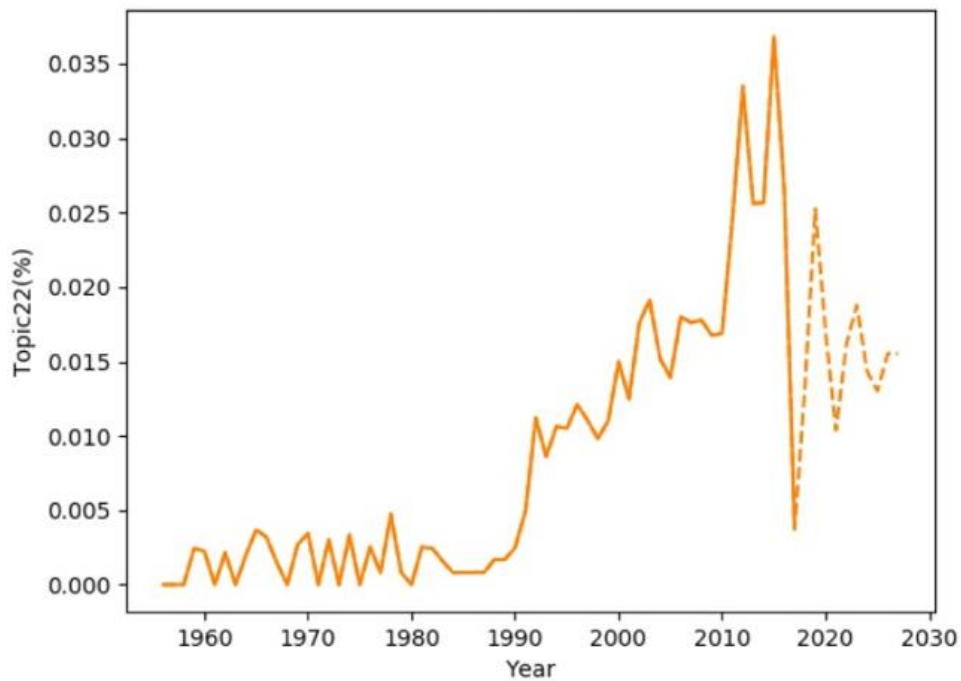

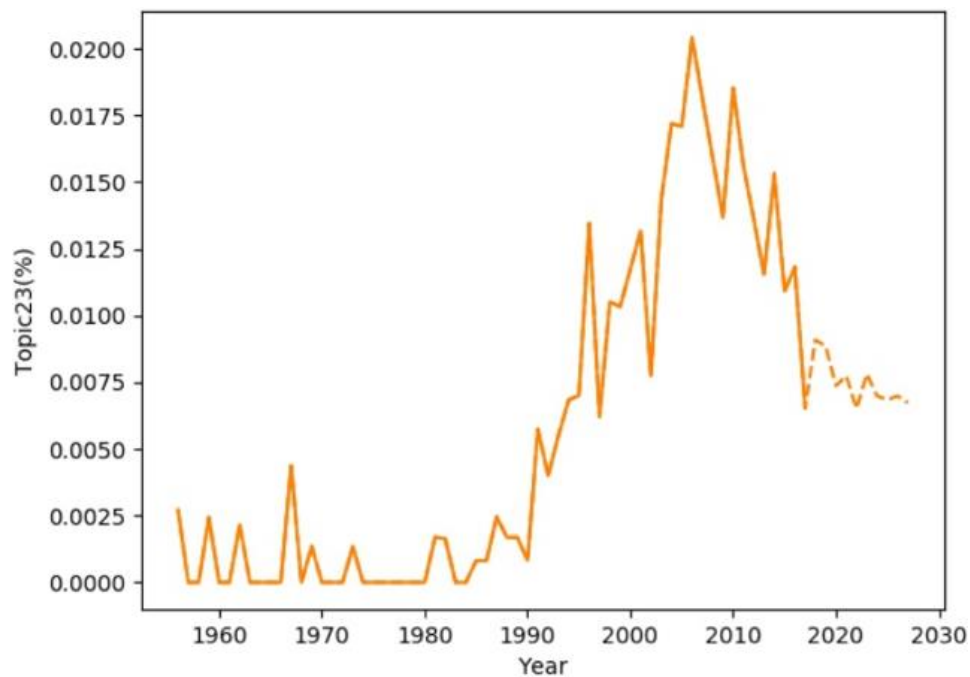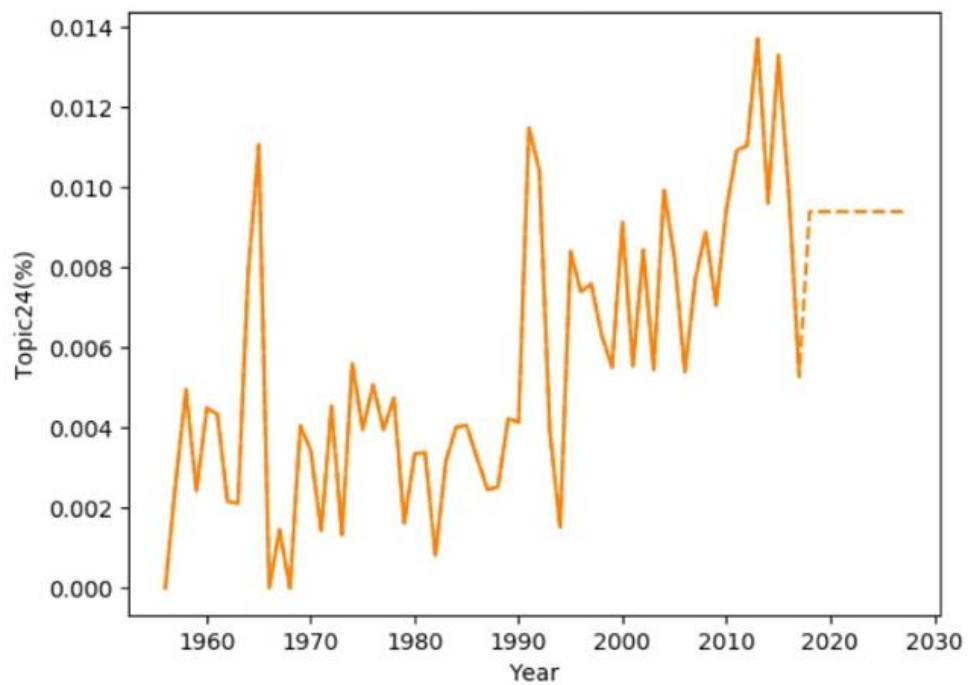

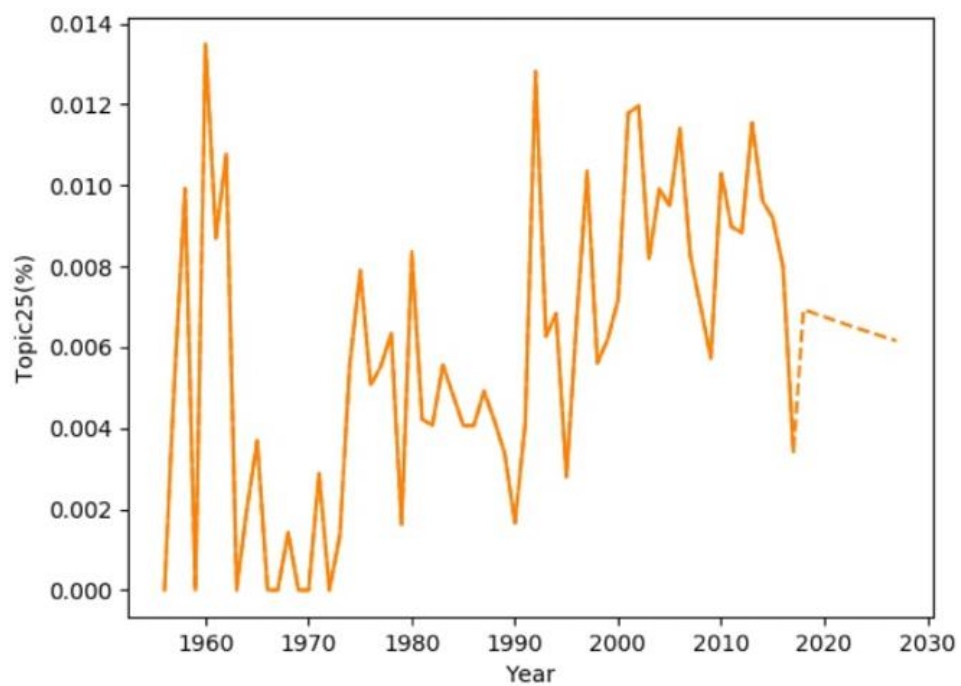

Supplement: S1 Fig — (PDF) [file pone.0199510.s001.pdf]
